# Supplementary material for: Mash: fast genome and metagenome distance estimation using MinHash
Source: Genome Biol. 2016 Jun 20;17:132. doi: 10.1186/s13059-016-0997-x (PMC4915045; doi:10.1186/s13059-016-0997-x)
Supplement: Additional file 1: Figure S1. — Absolute and relative error bounds for Mash Jaccard estimates given various sketch sizes. Figure S2. Error bounds for Mash distance estimate using k = 16 and k = 21 and various sketch sizes. Figure S3. Effect of k-mer and genome size on the Mash distance. Figure S4. Eukaryotic components of the RefSeq clustering, colored by taxonomic order. Figure S5. Plasmid and organelle components of the RefSeq clustering, colored by taxonomic species. Figure S6. Mash tree from Fig. 4 supplemented with five additional mammals. Figure S7. Mash clustering of all HMP and MetaHit sample assemblies. Figure S8. Raw COMMET output for the GOS dataset. Supplementary Note 1. Supporting data. Supplementary Note 2. Metagenomic heatmap R code. (PDF 8062 kb) [file 13059_2016_997_MOESM1_ESM.pdf]

Supplementary Materials for "Mash: fast genome and metagenome distance estimation using MinHash"

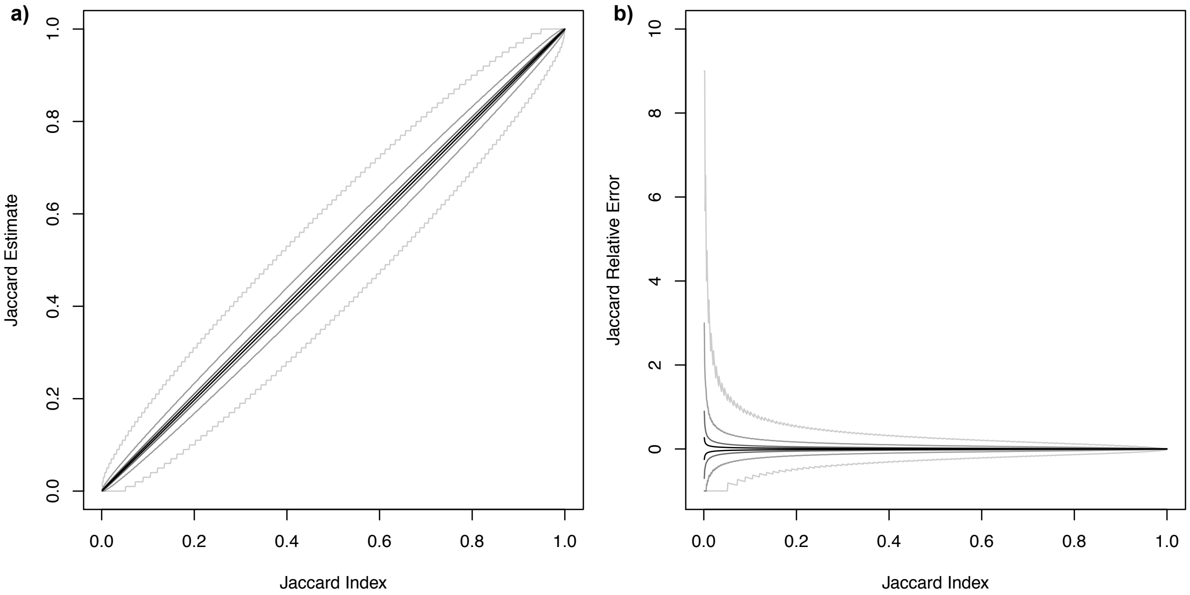

Figure S1. Absolute and relative error bounds for Mash Jaccard estimates given various sketch sizes. Increasing sketch sizes are progressively shaded from *s*=100 (light gray), *s*=1,000, *s*=10,000, and *s*=100,000 (black). Upper and lower bounds are drawn using the binomial inverse cumulative distribution function, with the same parameters from equation 8, such that for a given Jaccard index there is a 0.99 probability that the corresponding Jaccard estimate **(a)** or relative error **(b)** will fall within the bounds. These plots illustrate that relative error can grow quite large when estimating small Jaccard values. Thus, large sketch sizes are recommended when comparing divergent sequences with few shared k-mers. These plots only illustrate the error of the Jaccard estimate, and are independent of k-mer size. Supplementary Figures 2 and 3 show the relationship between Jaccard and the Mash distance, which does depend on k-mer size.

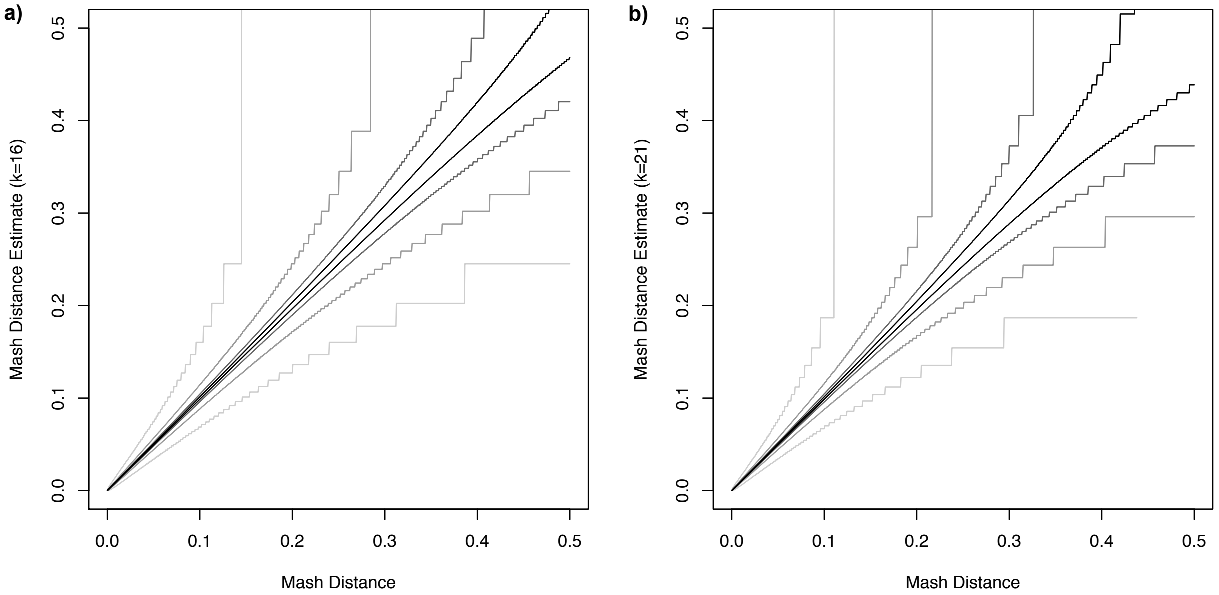

Figure S2. Error bounds for Mash distance estimate using *k*=16 and k=21 and various sketch sizes. Increasing sketch sizes are progressively shaded from *s*=100 (light gray), *s*=1,000, *s*=10,000, and *s*=100,000 (black). Upper and lower bounds are drawn using the binomial inverse cumulative distribution function, such that for a given Mash distance (and corresponding Jaccard index) there is a 0.99 probability that the corresponding Mash distance estimate will fall within the bounds for k-mer sizes of 16 **(a)** and 21 **(b)**. This plot illustrates that larger Mash distances require large sketch sizes to be accurately estimated. However, with a suitably large sketch size, accurate Mash distance estimation is possible across a wide range of values. Choosing a smaller k-mer size can also improve accuracy for divergent sequences, but k-mer choice also depends on genome size (Supplementary Figure 3).

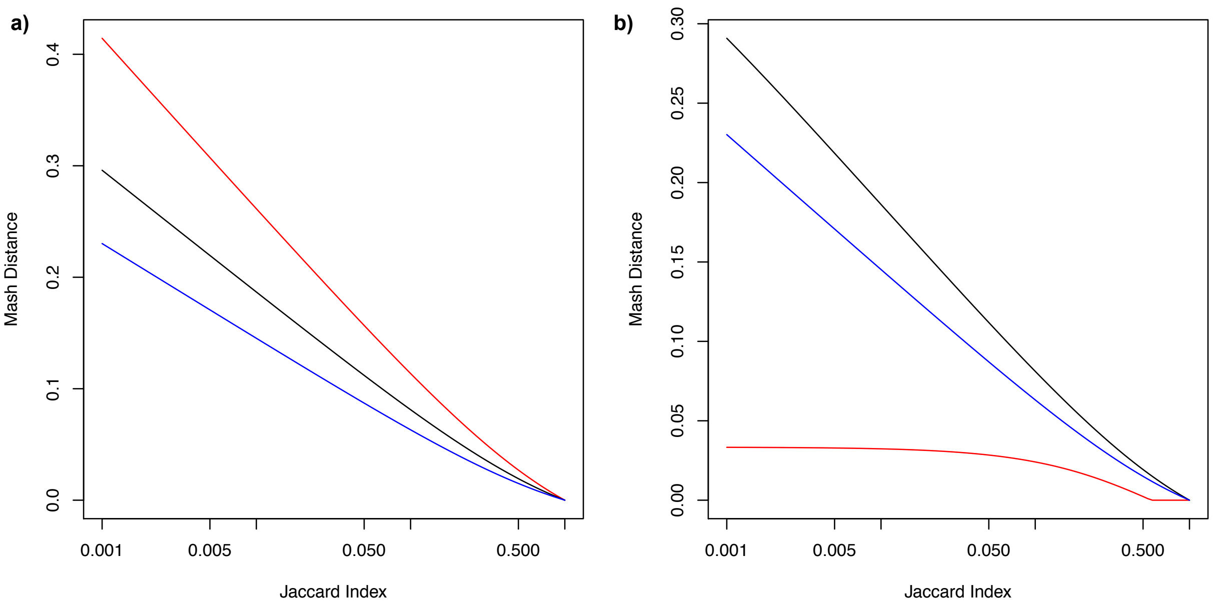

Figure S3. Effect of k-mer and genome size on the Mash distance. (a) The relationship between the Jaccard index and Mash distance for k-mer sizes of 15 (red), 21 (black), and 27 (blue) based on equation 4. The x-axis is log scale. For a fixed Mash distance (e.g. 0.2), larger k-mer sizes result in lower Jaccard scores because fewer, long k-mers are shared between divergent sequences. Thus, it can be helpful to use a small k-mer size to avoid the higher error that comes with small Jaccard values. This panel assumes all k-mers are unique. However, **(b)** illustrates the effect of non-unique k-mers and genome size, and adjusts the expected Mash distance based on the number of random k-mers that will be shared by chance between two 1 Gbp genomes. Here, the x-axis shows a hypothetical Jaccard index, assuming all k-mers are unique, but the y-axis shows the Mash distance accounting for such collisions. From equation 1 it is expected that two random genomes of this size will share many short k-mers by chance, leading to a nonzero expected Jaccard index (equation 5). This is seen in the curve for k=15 (red), for which the Mash distance never exceeds ~0.03, which matches the expected Mash distance between two 1 Gbp genomes for k=15. Equation 2 can be used to choose a more appropriate value of k. In this case, both k=21 (blue) and k=27 (black) largely eliminate random collisions and produce the expected curves. Generally, the smallest choice of k that eliminates most chance k-mer collisions is best, because it maximizes sensitivity without skewing the resulting Mash distance.

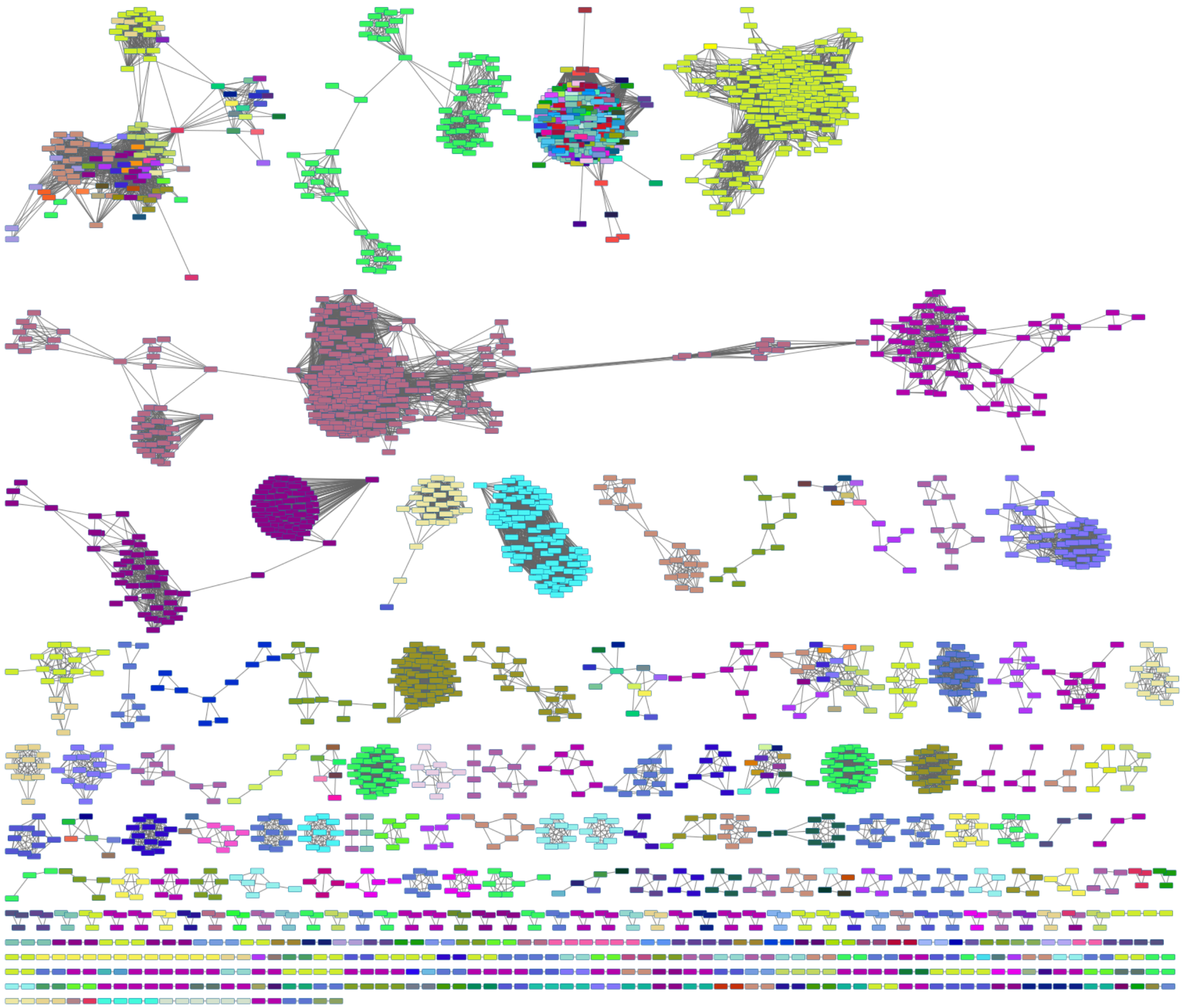

Figure S4. Eukaryotic components of the RefSeq clustering, colored by taxonomic order. Most well-defined clusters are fungi. The heterogeneous cluster at top, second from right, contains most large genomes (e.g. >1 Gbp in size). This over-clustering is a result of skewed Mash distances due to the small choice of *k*=16 used for the all-RefSeq clustering, which was targeted at microbial genomes. Using a larger value of *k* (e.g. 21) removes the distance skew and provides more accurate distance estimates for large genomes (e.g. Figure 4). Also, given that distinct eukaryotic species often have ANI values >95%, a lower Mash distance threshold would be required to separate this cluster by species.

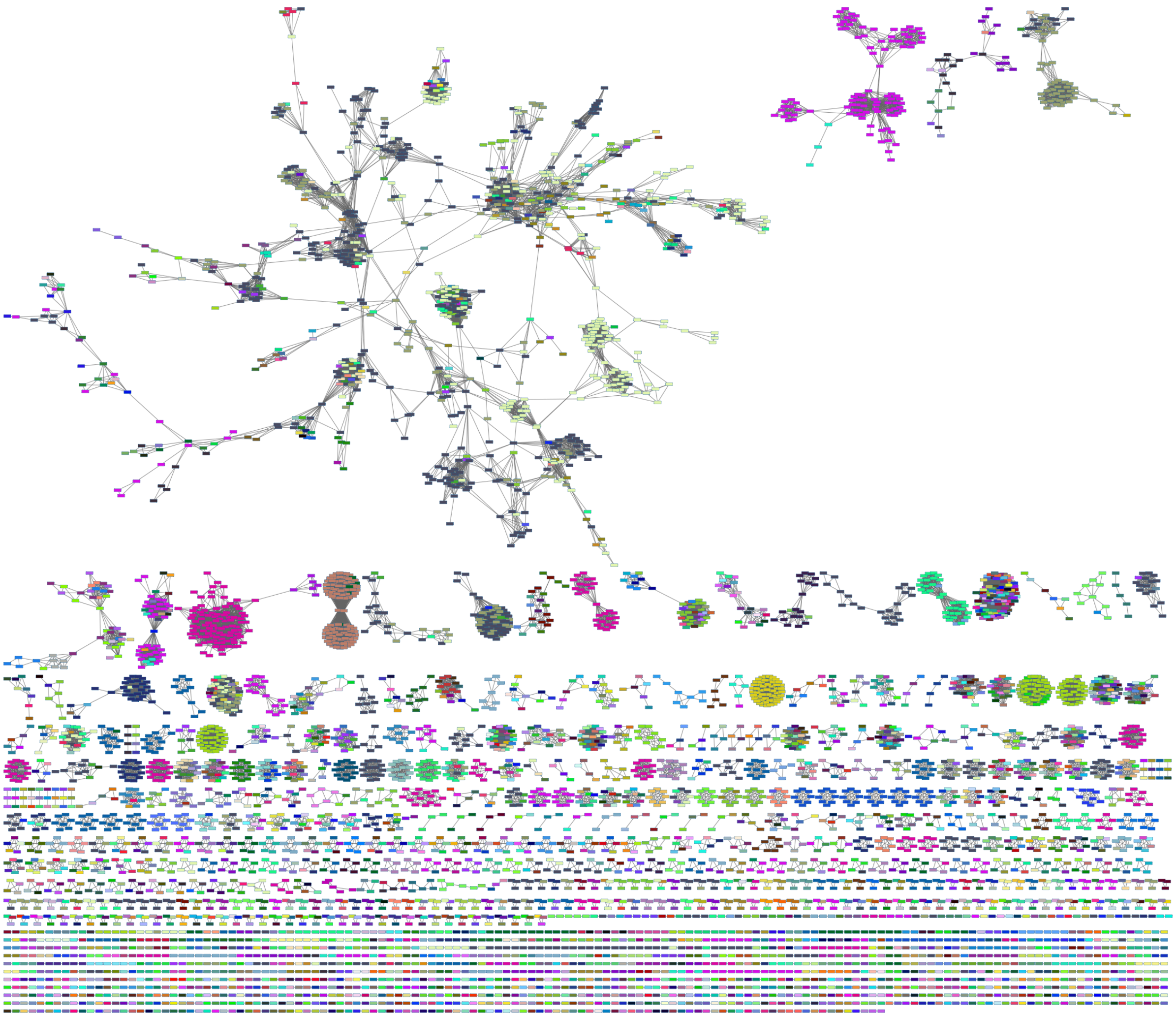

Figure S5. Plasmid and organelle components of the RefSeq clustering, colored by taxonomic species. Closely related plasmids are often species-specific, as illustrated by the uniform coloring in many of the components. However, the sprawling cluster at top left includes plasmids from many different species of Enterobacteriaceae.

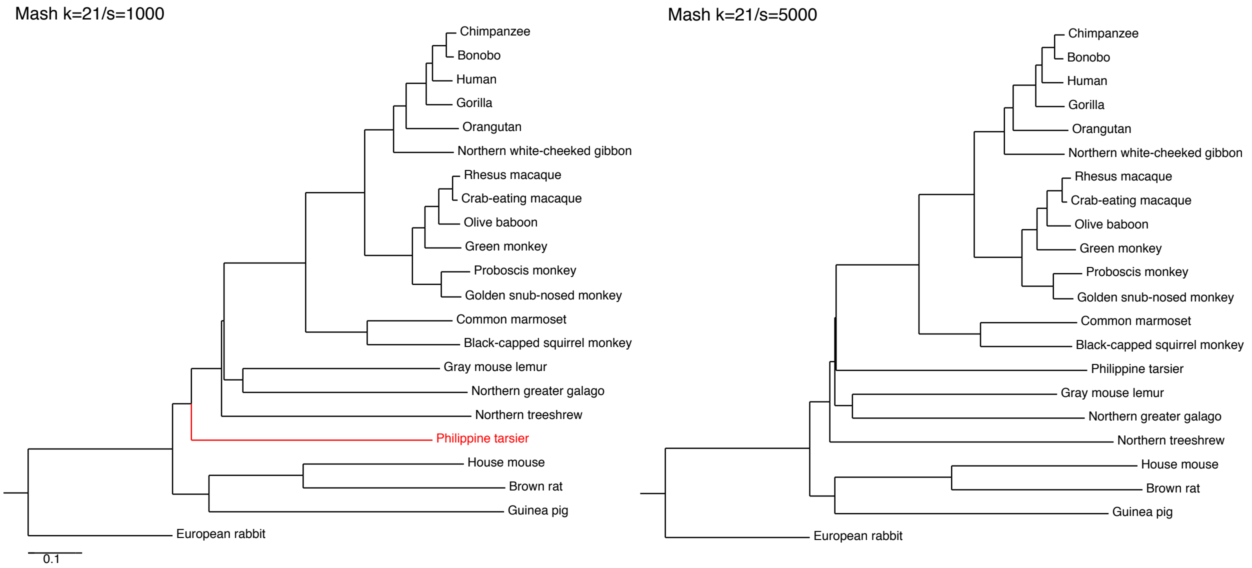

Figure S6. Mash tree from Figure 4 supplemented with five additional mammals.
Increased sketch sizes are needed to compensate for increased levels of divergence. With a default sketch size of 1,000 and k-mer size of 21, the inclusion of five additional genomes with increased divergence (treeshrew, mouse, rat, guinea pig, and rabbit) causes the tarsier genome to become misplaced (red). Increasing the sketch size to 5,000 corrects this misplacement.

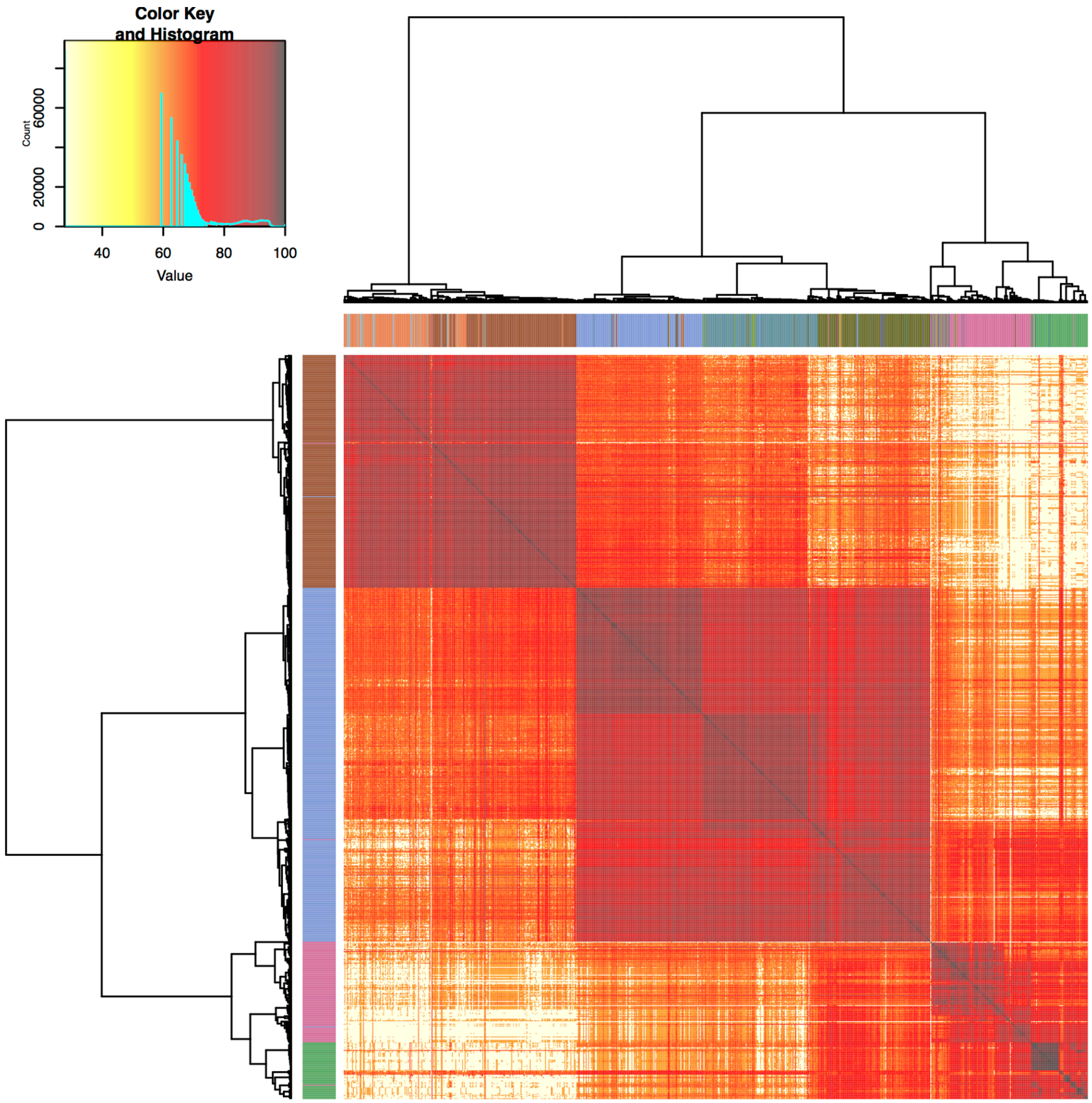

Figure S7. Mash clustering of all HMP and MetaHit sample assemblies. Color key is the same as that in Figure 5, with gross body site clustering on the left (e.g. skin, mouth) and sub-site clustering on the top (e.g. nares, tongue). A few outliers can be seen that fail to cluster with the main groups. Upon further inspection, it was found that these samples failed to pass the HMP QC requirements based on attributes that include mean contig and ORF density, human hits, rRNA hits, and data size. Thus, the Mash clustering supported the earlier HMP determination that these samples were outliers.

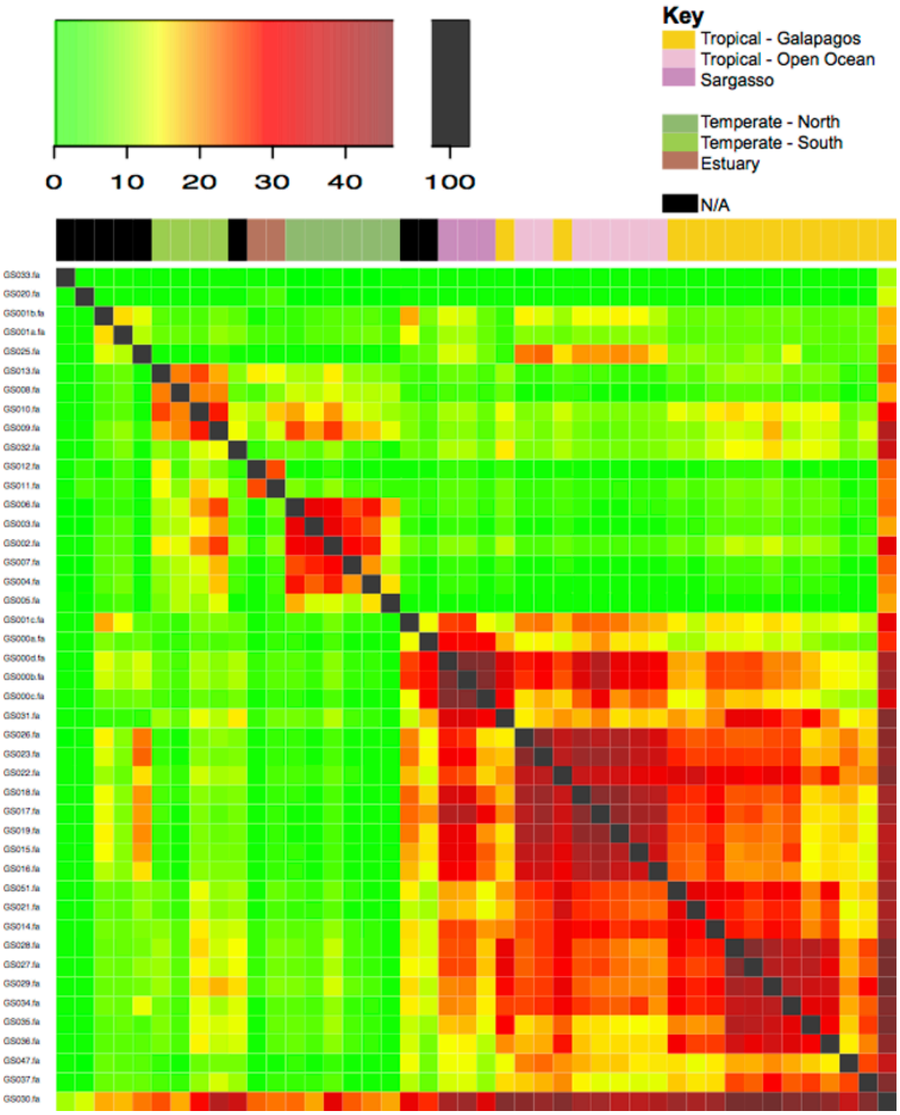

Figure S8. Raw COMMET output for the GOS dataset. An automatically generated COMMET plot for the GOS dataset. The same clustering is visible as in Figure 4 with a modified orientation and color palette.

Table S1. Names and accessions for the 17 primate and 5 mammal genomes.

Accession	Scientific name	Common name
GCF_000004665.1	Callithrix jacchus	Common marmoset
GCF_000409795.2	Chlorocebus sabaeus	Green monkey
GCF_000151905.1	Gorilla gorilla gorilla	Gorilla
GCF_000001405.28	Homo sapiens	Human
GCF_000364345.1	Macaca fascicularis	Crab-eating macaque
GCF_000002255.3	Macaca mulatta	Rhesus macaque
GCF_000146795.2	Nomascus leucogenys	Northern white-cheeked gibbon
GCF_000181295.1	Otolemur garnettii	Northern greater galago
GCF_000258655.1	Pan paniscus	Bonobo
GCF_000001515.6	Pan troglodytes	Chimpanzee
GCF_000264685.2	Papio anubis	Olive baboon
GCF_000001545.4	Pongo abelii	Orangutan
GCF_000769185.1	Rhinopithecus roxellana	Golden snub-nosed monkey
GCF_000235385.1	Saimiri boliviensis boliviensis	Black-capped squirrel monkey
GCF_000164805.1	Tarsius syrichta	Philippine tarsier
GCA_000772465.1	Nasalis larvatus	Proboscis monkey
GCF_000165445.1	Microcebus murinus	Gray mouse lemur
GCA_000181375.1	Tupaia belangeri	Tree shrew
GCF_000001635.24	Mus musculus	House mouse
GCF_000001895.5	Rattus norvegicus	Brown rat
GCF_000151735.1	Cavia porcellus	Guinea pig
GCF_000003625.3	Oryctolagus cuniculus	European rabbit

Supplementary Note 1. Supporting data.

The RefSeq Release 70 Mash sketch database and Escherichia accessions, ANI, Jaccard scores, and Mash v1.0 source code are available from http://mash.readthedocs.org/en/latest/data.html.

Supplementary Note 2. Metagenomic heatmap R code.

For COMMET, the default clustering method of complete was used, following the built-in R script. Clustering with the ward.D2 method did not significantly alter the sample clusters. Heatmaps were generated with the commands:

```
# read color key
key=read.table("key")
labels=key[,1]
labelColors=rgb(key[,2], key[,3], key[,4], maxColorValue=255)
bodySiteColors=rgb(key[,5], key[,6], key[,7], maxColorValue=255)

# read distance matrix
x = read.table("mash.ltbl");
y=x[,2:dim(x)[2]]
z = data.matrix(y)
z[is.infinite(z)]=0
```

```
rc = hclust(as.dist(z), method="ward.D2")

# convert to similarity
cr3 = data.matrix(y)
cr3=100-(cr3*100)

# define colors
n=100 # number of steps between 2 colors
mini=min(cr3[])
maxi=max(cr3[row(cr3)!=col(cr3)])
trueMax=max(cr3[])
q25=quantile(cr3[row(cr3)!=col(cr3)],0.25,1)
q50=quantile(cr3[row(cr3)!=col(cr3)],0.5,1)
q75=quantile(cr3[row(cr3)!=col(cr3)],0.75,1)

mini=max(q25-1.5*(q75-q25),0)
maxi=min(q75+1.5*(q75-q25),trueMax)
diff=maxi-mini

palette=colorRampPalette(c("lightyellow", "yellow", "red", "brown",
"grey23"))(n = 5*n-1)

 breaks=c(seq(mini,mini+diff/4-0.1,length=n), # for lightyellow
   seq(mini+diff/4,mini+diff/2-0.1,length=n), # for yellow
   seq(mini+diff/2,mini+3*diff/4-0.1,length=n), # for red
   seq(mini+3*diff/4,maxi-5,length=n), # for brown
   seq(from=maxi-5+0.1, to=trueMax, length=n))

library("gplots")
heatmap.2(cr3, Rowv=rev(as.dendrogram(rc)), Colv=as.dendrogram(rc),
labRow=as.matrix(labels), labCol=as.matrix(labels), scale="none",
distfun=as.dist, col=palette, ColSideColors=labelColors,
RowSideColors=bodySiteColors, trace="none", breaks=sort(breaks))
```
